# Change in Dengue and Japanese Encephalitis Seroprevalence Rates in Sri Lanka

**DOI:** 10.1371/journal.pone.0144799

**Published:** 2015-12-22

**Authors:** Chandima Jeewandara, Laksiri Gomes, S. A. Paranavitane, Mihiri Tantirimudalige, Sumedha Sandaruwan Panapitiya, Amitha Jayewardene, Samitha Fernando, R. H. Fernando, Shamini Prathapan, Graham S. Ogg, Gathsaurie Neelika Malavige

**Affiliations:** 1 Centre for Dengue Research, Department of Microbiology, Faculty of Medical Sciences, University of Sri Jayawardenapura, Nugegoda, Sri Lanka; 2 Department of Family Medicine, Faculty of Medical Sciences, University of Sri Jayawardanapura, Nugegoda, Sri Lanka; 3 MRC Human Immunology Unit, Weatherall Institute of Molecular Medicine, Oxford NIHR Biomedical Research Centre and University of Oxford, United Kingdom; 4 Department of Dermatology, Churchill Hospital, Oxford, United Kingdom; Institut Pasteur of Shanghai, CHINA

## Abstract

**Background:**

Sri Lanka has been affected by epidemics of dengue infections for many decades and the incidence and severity of dengue infections have been rising each year. Therefore, we investigated the age stratified seroprevalence of dengue infections in order to facilitate future dengue vaccine strategies. In addition, since the symptomatic dengue infections have increased during the past few decades, we also investigated the possible association with Japanese Encephalitis Virus (JEV) antibody seropositivity with symptomatic dengue in a community cohort in Sri Lanka.

**Methods:**

1689 healthy individuals who were attending a primary health care facility were recruited. Dengue and JEV antibody status was determined in all individuals and JEV vaccination status was recorded.

**Results:**

1152/1689 (68.2%) individuals were seropositive for dengue and only 133/1152 (11.5%) of them had been hospitalized to due to dengue. A significant and positive correlation was observed for dengue antibody seropositivity and age in children (Spearmans R = 0.84, p = 0.002) and in adults (Spearmans R = 0.96, p = 0.004). We observed a significant rise in the age stratified seroprevalence rates in children over a period of 12 years. For instance, in year 2003 the annual seroconversion rate was 1.5% per annum, which had risen to 3.79% per annum by 2014. We also found that both adults (p<0.001) and in children (p = 0.03) who were hospitalized due to dengue were more likely to be seropositive for JEV antibodies. However, 244 (91.4%) of adults who were seropositive for JEV had not had the JEV vaccine.

**Conclusions:**

Dengue seroprevalence rates have risen significantly over the last 12 years in Sri Lanka, possibly due to increased transmission. As individuals who were hospitalized due to dengue were more likely to be seropositive for JEV, the possibility of cross-reactive assays and/or of JEV infection on immunity to the DENV and clinical disease severity should be further investigated.

## Background

Dengue infections are one of the major public health problems in Asia and in Latin America, with an estimated burden of 390 million infections per year [1]. The annual burden of dengue is estimated to be higher than the global burden of 17 other disease conditions of public health importance [2]. The global disability adjusted life years (DALYs) due to dengue have increased by 17% from 1990 to 2013 with DALY for dengue in year 2013 reported as being 1,142.7 thousands years [3]. Currently, there are no effective drugs for treatment of acute infection, nor a licensed vaccine for prevention. However, a candidate dengue vaccine has completed a phase 3 clinical trial [4] and many other dengue vaccine investigators are currently planning large scale multi-centre clinical trials [5]. Therefore, in order to determine future dengue vaccine strategies and for initiation of dengue vaccine clinical trials, it would be important to determine the seroprevalence of dengue infections in various age groups in Sri Lanka.

Sri Lanka has been affected by epidemics of dengue infections for the past 3 decades and the incidence and severity of these epidemics is increasing [6]. The incidence of dengue has been particularly high since 2009, and the facilities available in most resource-poor hospitals in Sri Lanka have been under pressure. Dengue infections are hyperendemic in urban Sri Lanka and 50% of children <12 years were found to be seropositive for dengue [7]. However, the seroprevalence rates were shown to be lower in suburban populations, as a study among children in a suburban area in Sri Lanka in 2003, showed that only 34% of children had dengue virus (DENV) specific IgG antibodies [8]. As the epidemiology of dengue infections has changed significantly in Sri Lanka during the past decade, it would be important to determine changes in seroprevalence rates to further understand epidemiological trends and to plan dengue vaccine trials.

Although the Sri Lankan population has been exposed to the virus for decades and small outbreaks of dengue infection were reported in the 1960s [9, 10], regular large outbreaks of dengue infections and more severe forms of dengue were more common from 1989. Since then the number of cases of dengue infection has increased with the infection now spreading to all parts of Sri Lanka [11]. An increase in the number and severity of dengue infections have been seen globally, possibly due to more intense transmission [12]. However, it is also possible that the rise in the incidence of dengue could be due to increase in severity, which would in turn lead to an increase in the number of diagnosed symptomatic dengue infections compared to asymptomatic dengue.

Although secondary dengue infection is currently a well-known risk factor for development of severe dengue, more recent studies have shown that the presence of pre-existing antibodies to the Japanese Encephalitis virus (JEV) were associated with a greater risk of developing a symptomatic dengue infection [13]. The JEV and the DENV, which are both transmitted by mosquitoes are highly endemic in the South Asian and the South East Asian regions [14]. Many children living in JEV and DENV endemic countries receive the JEV vaccine and immune responses to the vaccine or natural infection could potentially modulate immune responses to subsequent dengue infection. Due to the similarity of the JEV and the DENV, both antibody and T cell responses have been shown to cross react with each other [15–17]. In Sri Lanka, immunization against the JEV started in a phase by phase basis, and since 1988 was subsequently included in the national routine immunization program [18]. Since the JEV vaccine was first introduced to Sri Lanka in 1988, which was the year before Sri Lanka experienced regular epidemics of dengue infections, it would be important to determine if JEV seropositivity was associated with an increased risk of symptomatic dengue.

In this study, we initially determined the age stratified dengue seroprevalence rates in a large cohort of adult and children representative of the suburban population in Colombo, Sri Lanka. In order to determine the changes in DENV seroprevalence rates, we compared the rates of children in this study with one of our previous studies done 12 years ago, which was done in the same geographical region. We also proceeded to determine the JEV seropositivity and JEV vaccination status of these individuals in order to determine if JEV seropositivity either due to natural infection/immunization or due to the presence of cross reactivity antibodies was associated with hospitalization due to dengue infection.

## Methods

### Study population

The study included 1689 healthy individuals attending the Family Practice Centre, which is a primary health care facility of the University of Sri Jayewardenepura, Sri Lanka, providing community health care to over 2000 families living in the suburban areas of the Colombo district. Individuals registered at the Family Practice Centre between the ages of 6 to 80 years were invited to participate in this study and were recruited in the latter part of 2013 and early 2014, following informed written consent. In the case of children, informed written consent was obtained from the parent or guardian. 1081(64%) of the individuals were over 16 years of age. The individuals who were recruited for this study were representative of the population living in this broader geographical region and representative of a suburban population in Colombo.

In addition to their detailed primary care records, an interviewer administered questionnaire was used to record demographic details and whether the participants had been admitted to hospital in the past due to a febrile illness and in such cases if it was due to dengue infection and severity of illness. Children were defined as individuals aged ≤16 years of age and adult as individuals >16 years of age. According to classification of WHO guidelines 2011 [12], of the 133 individuals who were hospitalized, 72/80 adults (individuals aged >16 years) were diagnosed as having DHF and 8/80 adults diagnosed as having DF. 53/608 children (individuals who were ≤ 16 years) who were hospitalized due to a febrile illness were diagnosed as having DHF. This information was obtained from the diagnosis card given to the patient when discharged from hospital. All those who reported to have past dengue infection, were laboratory confirmed by either being NS1 positive and or DENV IgM/ IgG positive at the time of infection. These data were obtained from the diagnosis card given by the hospitals on discharge.

### Ethics statement

Ethical approval was granted by Ethical Review Committee of the University of Sri Jayewardanapura, Sri Lanka.

### Comparison of the changes in DENV seroprevalence in year 2003 with the current study

In one of our previous studies done in year 2003, we had determined the DENV seroprevalence rates of 313 children (aged ≤16 years) in the same geographical region as the present study, using the same DENV IgG ELISA assay (Panbio Dengue IgG Indirect ELISA- Australia) to determine seropositivity [8]. During a period of 12 years, the epidemiological patterns of DENV infections changed dramatically in Sri Lanka, with a more than 10 times rise in the number of DENV infections reported from the same region. As there was a dramatic rise of the incidence of dengue, we proceeded to determine the effect it had on DENV seroprevalence rates, hypothesizing that there would be an equally dramatic rise in the DENV seroprevalence rates than our previously observed rates of 34% [8]. Therefore, as we had determined the seroprevalence rates of children aged 6 to 16 in the earlier study, in order to compare the changes in seroprevalence, we calculated the changes in seroprevalence rates in the same age group in the present study.

### JE vaccination status

Vaccination records were also obtained from all participants to determine if they had received the Japanese encephalitis vaccine (JEV). None of the participants had a clinically diagnosed JEV infection in the past or encephalitis like infection. As the JEV killed vaccine was used till 2009, and the live attenuated vaccine given to the majority of children from year 2009 onwards, individuals who had received the JEV vaccine after year 2009 had either received the killed JEV vaccine or the live attenuated vaccine. In Sri Lanka those who received the live vaccine, only received one dose of the vaccine at either 1 year or at 9 months.

In our cohort of children ≤16 years of age, 565/608 (92.9%) had received the JE vaccine. 311/565 (55%) had received live vaccination for JE at the age of 1 year or less and 203/565 (35.9%) children had received a four dose killed vaccine schedule from either Nakayama strain or Beijing strain of the killed JE vaccine given at the age of 1 to 10 years. 51/565 (9%) children did not complete the killed vaccine schedule. Only 23/1081(2.13%) adults (aged >16 years) had received a JE vaccination in the study population. All adults who received the JE vaccine had received a killed vaccine schedule.

### Determining dengue and JE seropositivity

JE Detect^TM^ IgG ELISA (InBios International USA) was used for the detection of antibodies in human serum to determine the presence of JEV-specific IgG antibodies. Calculation of the immune status ratio (ISR) done according to the manufacturers’ instructions and accordingly an ISR of > 5 was considered positive; an ISR of 2–5 equivocal and an ISR of < 2 negative. All individuals tested for indirect dengue IgG capture ELISA (Panbio) for the qualitative detection of IgG antibodies to DENV antigen serotypes (1, 2, 3, 4) in serum. PanBio units were calculated according to the manufacturer’s instructions and accordingly, PanBio units of > 11 were considered positive, 9–11 was considered equivocal and < 9 was considered negative.

### Statistical Analysis

PRISM version 6 was used in statistical analysis. As the data were not normally distributed, differences in means were compared using the Mann-Whitney U test (two tailed). To compare means of three or more variables, Kruskal-Wallis test was used. Chi Square tests, Chi square for trend or the Fisher’s exact test was used to determine the p value. Correlation of the antibody responses to age, symptomatic dengue infection and non-severe dengue infection were done using Spearman rank correlation. For comparison of the age stratified seroprevalence rates during 2003 and 2014, the Wilcoxon matched pairs sign ranking test was used. The annual seroconversion rates were calculated by the change in seroprevalence in each age group in children <16 years of age and was derived from the value given for the slope of the linear regression analysis.

## Results

Of the 1689 individuals recruited from the community, 608 (36%) were children ≤16 years of age, and 1081 (64.0%), were adults. Of the 608 children, 346 (56.9%) of them were males and 262 (43.1%) were females. Of the 1081 adults, 484 (44.7%) were males and 598 (55.3%) were females. The mean age of the adults was 45 years (95% CI: 44–47 years) and the mean age of the children was 8.8 years (95% CI: 8.6–9.1 years). 397/1689 (23.5%) individuals were seronegative for anti DENV antibodies. 1152/1689 (68.2%) were seropositive and 8.3% showed an equivocal response for dengue and only 133/1152 (11.5%) of them had been hospitalized to due to dengue. Since the other seropositive individuals had never been hospitalized for a febrile illness in the past, they were considered to have had a past mild or subclinical dengue infection.

### Age stratified seroprevalence rates for dengue

**Fig 1:**
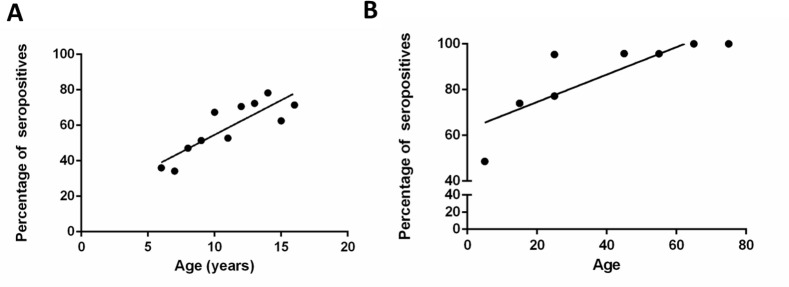
Seroprevalence of dengue infections. A: Age stratified seroprevalence of DENV-specific antibodies in children. B: Age stratified seroprevalence of DENV-specific antibodies in adults.

308/608 (50.7%) children were seropositive for anti-DENV antibodies and 982/1081 (90.8%) adults were seropositive for anti-DENV antibodies. A significant and positive correlation was observed for dengue antibody seropositivity and age in children (Spearmans R = 0.84, p = 0.002) (Fig 1A and Table 1) as well as in adults (Spearmans R = 0.96, p = 0.004) (Fig 1B and Table 1).

**Table 1:** Age stratified detection of anti-DENV antibodies.

Age in years	Seropositive N (%)	Seronegative N (%)	Equivocal N (%)	Total
<7	75 (34.4)	143 (65.6)	0(0)	218
8–10	117 (52.9)	99(44.8)	5 (2.3)	221
11–13	64(64.6)	34(34.3)	1 (1.0)	99
14–16	43(70.5)	17(27.9)	1 (1.6)	61
17–24	83 (76.9)	25(23.1)	0	108
25–34	152(77.2)	45(22.8)	0	197
35–44	206 (95.4)	10 (4.6)	0	216
45–54	229(95.8)	8 (3.3)	2 (0.8)	239
55–64	199(95.7)	9(4.3)	0	208
65–74	96(100)	0 (0)	0	96
>75	17(100)	0 (0)	0	17

### Changes in dengue seroprevalence rates of dengue over a 12 year period

**Fig 2:**
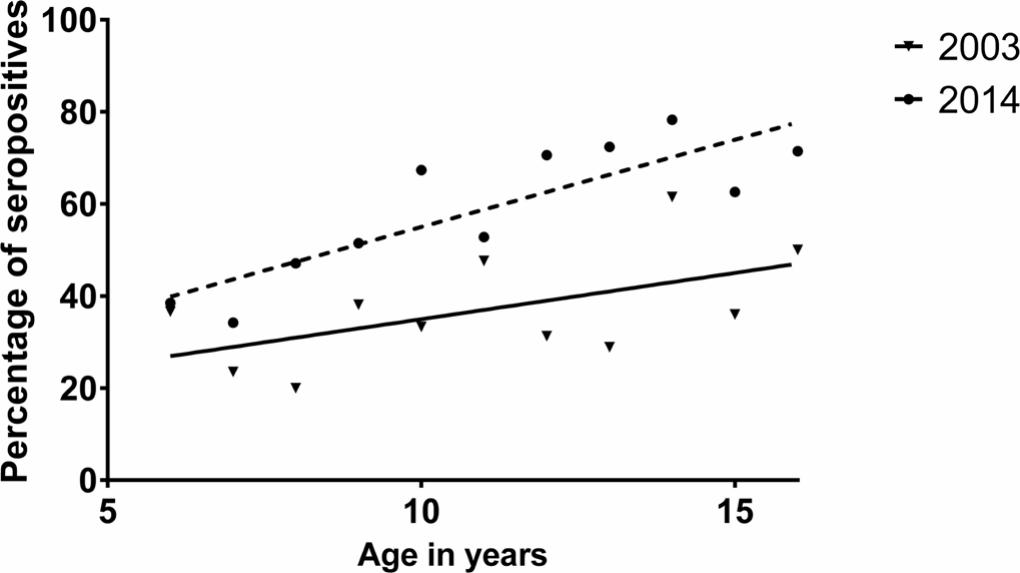
Comparison of the age stratified seroprevalence rates in children over a period of 12 years. The age stratified seroprevalence rates were compared in children aged 6 to 16 years in 2003 and 2014.

Although sporadic cases of DHF were seen in Sri Lanka for decades, epidemics of dengue infections only occurred since 1989. Since then the number of cases in each epidemic has been dramatically increasing. In 2003, we evaluated the dengue seroprevalence rates in children in the same geographical location. Therefore, we compared the differences in the age stratified seroprevalence of dengue over a period of 12 years. We found that the age stratified seroprevalence rates of dengue had significantly increased (p<0.001) (Fig 2). While the annual seroconversion rate in 2003 was 1.5±0.89% per annum, in 2014 the annual seroconversion had risen to 3.79 (±0.79)% per annum. These data support the higher incidence of dengue transmission in the community over the last twelve years.

### Association of f JE specific antibodies with age and vaccination status

In order to investigate if JEV vaccination was likely to be associated with JEV seropositivity in children, we investigated if children who received the JEV were more likely to be seropositive for JEV, even many years following vaccination. As expected we found that there was a statistically significant association (p = 0.04) between the presence of JEV specific antibodies and JEV vaccination (Table 2). However, only 143 (25.3%) of those who received the JEV vaccine were seropositive, whereas 297 (52.6%) were seronegative and 125 (22.1%) showed an equivocal response. Although only 23 (2.1%) of adults (individuals aged >16 years) were immunized for JEV, 267 (24.7%) had detectable JEV specific antibodies.

**Table 2:** Association of JE antibody positivity with past hospitalization due to dengue, JE vaccination status and the type of JE vaccine given in children.

Characteristic	JE antibody positiveN (%)	JE antibody negativeN (%)	JE antibody EquivocalN(%)	Total	P value(analyzed by Chi-square)
**Dengue severity**					
Past hospitalized dengue	30 (56.6)	5 (9.4)	18 (33.9)	53	0.03
Mild/asymptomatic dengue	105 (41.0)	57 (22.3)	120 (46.9)	256	-
**JE vaccination status**					
JE vaccine given	143 (25.3)	297 (52.6)	125 (22.1)	565	0.04
Not given	5 (11.6)	25 (58.1)	13 (30.2)	43	-
**Type of JE vaccine given**					
Killed	73 (35.8)	76 (37.3)	55 (26.9)	204	<0.001
Live	52 (16.7)	201 (64.6)	58 (18.6)	311	-

**Fig 3:**
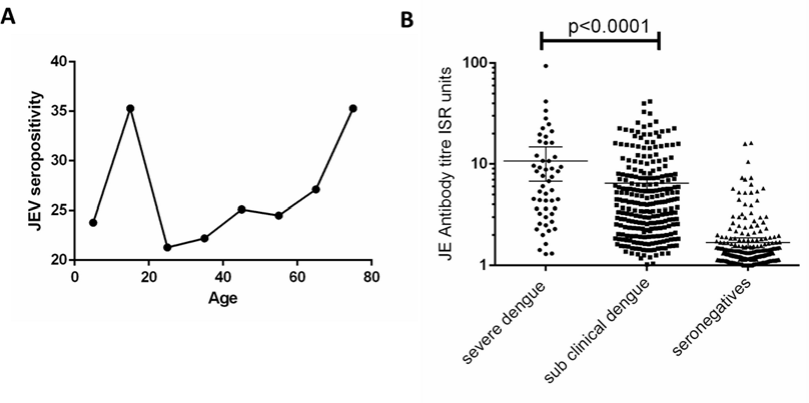
Association of JEV antibody seropositivity with age and severity of past dengue infection. 3A: Association of JEV antibody seropositivity with age. 3B: JE antibody titre of individuals who were hospitalized due to dengue (severe dengue), those who had mild/asymptomatic dengue (subclinical dengue) and dengue seronegative individuals.

Since the presence of JEV-specific antibodies could wane with time since vaccination, we next proceeded to determine JEV seropositivity and time since vaccination. All children had received the vaccine at either 9 months of age along with the measles vaccine (live attenuated vaccine), or between 12–15 months. However, we found that JEV antibody seropositivity significantly and positively correlated with age (Spearman’s R = 0.68, p = 0.007) (Fig 3A). For instance, although at 7 years 12.3% of children were seropositive for JEV, at 16 years it was 42.6%.

We next determined if the type of vaccine given influenced the proportion of those who remained persistently seropositive to JEV. As 51 children who initially received the JEV killed vaccine, did not complete the vaccination schedule, they were excluded from the analysis. We found that those who had received the killed JEV were significantly more likely (p<0.001) to be seropositive to JEV when compared to the live JEV vaccine (Table 2). For instance, 73 (35.8%) of those who received the killed vaccine were seropositive to JEV and 55 (26.9%) had an equivocal response, whereas only 52 (16.7%) of those who received the live vaccine were seropositive and only 58 (18.6%) had an equivocal response.

### JEV antibody seropositivity and association with hospitalization due to dengue

As it was recently shown that symptomatic dengue infection was more frequent among children who had detectable antibodies to the JEV and as JEV antibody positivity was shown to associate with symptomatic dengue in this study [13], we proceeded to determine if JEV seropositivity either due to natural infection/immunization or due to the presence of cross reactivity antibodies was associated with hospitalization due to dengue infection.

We found that a statistically significant association (p = 0.03) was seen between JEV antibodies being equivocal to positive and being hospitalized due to dengue (Table 2). For instance, 56.6% of children who had been hospitalized due to dengue were seropositive for JEV, while only 41% of those who had mild/asymptomatic dengue were seropositive. In addition, only 9.4% of those who were hospitalized due to dengue were seronegative for JEV, whereas 22.3% of those who had mild/asymptomatic dengue were seronegative. Although only 23 (2.1%) of adults (individuals aged >16 years) were immunized for JEV, 267 (24.7%) had detectable JEV specific antibodies. However, as observed with children, a statistically significant association (p<0.001) was seen from JEV antibodies being equivocal to positive with being hospitalized due to dengue. For instance, 34 (42.5%) of adults who had been hospitalized due to dengue had JEV specific antibodies, whereas only 233 (23.3%) individuals who had mild/asymptomatic dengue had JEV specific antibodies. However, 244 (91.4%) of the JEV seropositive adults who had JEV-specific antibodies were not immunized for the JEV.

We also found that children and adults who were hospitalised due to dengue had significantly higher (p<0.0001) JEV antibody titres (mean = 10.8, 95% CI 6.7–14.8) than individuals who were seropositive for dengue but were never hospitalised due to dengue infection (mean = 6.5, 95% CI 5.6–7.3) (Fig 3B).

### Cross reactivity between JEV and DENV IgG antibodies

**Fig 4:**
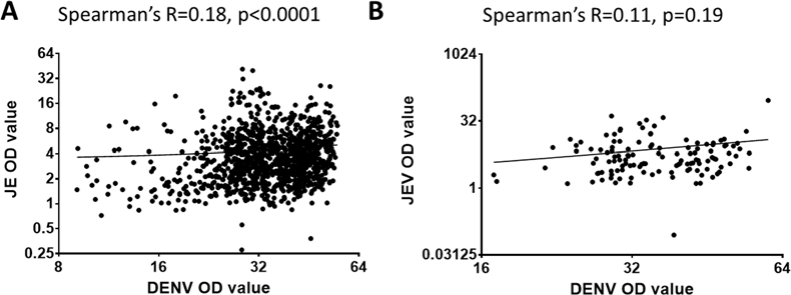
Association of JEV antibody optic density (OD) values with DENV antibody OD values. 4A: Correlation of JEV IgG antibody OD values and DENV IgG OD values in individuals who had mild/asymptomatic dengue. 4B: Correlation of JEV IgG antibody OD values and DENV IgG OD values in individuals who were hospitalized due to dengue.

Flavivirus antibodies are known to be highly cross reactive in nature and are known to give false positive responses in antibody detection assays [16, 19, 20] and the higher JEV seropositivity in adults who were hospitalized due to dengue, could possibly be due to higher cross-reactive DENV antibody titres (and therefore JEV antibody titres). Therefore, we determined the association between the optic density (OD) values for DENV and JEV in those with hospitalized dengue and those who had asymptomatic/mild dengue. The Spearmans R value was 0.18 (p<0.0001) for OD values between DENV and JEV in those who had a mild/asymptomatic infection (Fig 4A). However, the Spearmans R value was 0.11 (p = 0.19) for OD values between DENV and JEV in those who had been hospitalized due to dengue (Fig 4B). In addition, there was no correlation between the OD values of DENV IgG in individuals who were hospitalized due to dengue (mean 36.7, SD±8.7), and those who had mild/asymptomatic dengue (mean 35.4, SD±9.7), (Spearmans R = 0.07, p = 0.42).

## Discussion

In this study we have determined the dengue seroprevalence rates, changes in seroprevalence rates over time and association of JEV vaccination and seropositivity with hospitalization when infected with the DENV in a large suburban community cohort of individuals from Sri Lanka. We observed that by 16 years, 70.5% of the population was seropositive for dengue, which rose to 95.4% by 44 years of age. Epidemics of DHF have been increasing in frequency in Sri Lanka with the number of cases rising with each epidemic. For instance, in year 2003, when we determined the seroprevalence rates of dengue in the same community, the total number of cases reported from Colombo during that year was 1010 [21]. In year 2014, when we again determined the seroprevalence rates, a total number of 14,669 cases were reported, which is more than a 10 fold rise in the incidence. Therefore, as expected we observed a significant rise in the age stratified seroprevalence rates in children over a period of 12 years. In 2003 the annual seroconversion rate was 1.5% per annum, which had risen to 3.79% per annum by 2014. Although a rise in the seroconversion rates would be compatible with the higher incidence of dengue, this does not appear to reflect a more than 10 fold rise in the number of cases being reported by hospitals. In fact, studies done in more urban areas in Colombo have shown that there has not been much change in the seroprevalence rates among children during a similar time period [22, 23]. Therefore, the higher number of reported cases of dengue could be due to better reporting or increase in severity of clinical disease when infected with the DENV.

The DENV and JEV are both flavi-viruses which are closely related and co-circulate in the same geographical region. Therefore, pre-existing immune responses JEV could modulate the immune response to the DENV and thus influence the outcome of infection. In fact, it was shown in a recent study, that symptomatic dengue was more frequent in children who had detectable JEV specific antibodies [13]. Similar to the findings of Anderson et al, we too found that JEV specific antibodies were significantly more likely to be detected from those who had been hospitalized due to dengue, when compared to those who had mild/asymptomatic dengue [13]. However, as we used a JEV-specific IgG ELISA for detection of JEV specific antibodies, there is a high possibility of the occurrence of false positives due to highly cross reactive nature of the antibody responses to these two viruses. Therefore, it is possible that individuals who were hospitalized due to dengue (probably due to a secondary dengue infection), are more likely to have JEV specific IgG antibodies as infection with several DENV could boost a cross-reactive anti JEV antibody response. However, a recent study from Sri Lanka showed that children who developed symptomatic dengue were more likely to have broadly neutralising antibody responses when compared to children who developed asymptomatic dengue [24]. Children with asymptomatic dengue were shown to have predominantly mono-specific neutralising antibodies, which suggest that their neutralising antibodies were mostly specific to the past infecting serotype [24]. Therefore, the presence of neutralising antibodies which react with other DENV serotypes and possibly also other flavi-viruses such as JEV appears to associate with a higher risk of symptomatic dengue.

JEV vaccination was initiated in Sri Lanka in a phase by phase basis in year 1988. Although DENV infections were shown to be endemic in Sri Lanka with many adults having antibodies that react to the DENV, dengue infections causing regular epidemics of DHF only occurred in year 1989 [11]. Although it is likely that it is a pure co-incidence that epidemics of DHF started to occur in Sri Lanka in 1989 following the commencement of JEV vaccination in 1988, the possible association between JEV seropositivity and dengue disease severity should be further investigated as JEV is routinely administered to young children in many DENV endemic countries

In this study, we found that JEV antibody seropositivity increased with age and that 24.7% of adults who had not received the JEV vaccine had JEV-specific IgG antibodies. Although it was expected that JEV-antibody seropositivity is likely to decline with age, due to possible waning of immunity, as reported in other JEV endemic countries, we found that there was a significant and positive correlation between the age and seropositivity [14, 25, 26]. Since there is a high cross reactivity between IgG antibodies specific to JEV and the DENV, it is possible that individuals who are sequentially infected with many serotypes of DENV develop more broadly cross reactive antibodies that give false positive results for JEV IgG ELISA [14, 27, 28]. However, it is also possible that individuals are frequently exposed to the JEV resulting in JEV IgG antibody seropositivity. JEV is known to cause symptomatic infection in only one in 2 million infections, in vaccinated populations [29]. A study in Vietnam indeed did show that a significant proportion of the population had been infected with the JEV asymptomatically [14]. Therefore, in order to develop more effective and safer dengue vaccines, it would be crucial to understand if and how immune responses to the JEV modulate subsequent immune responses to the DENV either following vaccination or after natural infection.

Currently two types of vaccines are available for the prevention of JEV. The JEV live attenuated vaccine, which is cell culture derived, contains an attenuated neuro invasive strain of the JEV (SA-14-14-2) [30]. 100% of children who were between 2–5 years and 96% of children who were 12–24 months, and were given the JEV live attenuated vaccine were found to have seroprotected 28 days after vaccination [31]. However, JEV seropositivity has been shown to reduce over time to 96% in the 2–5 year old age group and to 84% in the 12–24 month age group 1 year following vaccination [31] and to 63.8% after 5 years when JEV specific antibodies were measured by the plaque reduction neutralization assay [32]. The JEV inactivated vaccine has also shown comparable efficacy to the live vaccine, although a higher rate of adverse effects have been reported [33, 34]. Although JEV seropositivity up to 5 years since vaccination has been studied, long term seropositivity following vaccination has not been well documented. In this study 92.6% of the children had been given the JEV live or killed vaccine and 25.3% had JEV specific antibodies many years following vaccination. It is likely to that we detected a lower JEV IgG seropositivity rate, as the assay we used was found to have a poorer sensitivity that the plaque reduction neutralization test [35]. However, Anderson et al who used plaque reduction neutralization assays (PRNTs), which is a more functional method than ELISA, to detect JEV-specific IgG antibodies in children aged 5–10 years, reported JEV antibody seropositivity of 46% in those who had received the vaccine [13]. In addition, an unpublished study carried out by the Ministry of Health, Sri Lanka reported, only 40% of children had a PRNT ≥10 one year following vaccination with the live JEV [36]. Therefore, although it appears that JEV seropositivity rates following 5–10 years of vaccination are generally low, the JEV seropositivity levels in our study was particularly low due to the method used for detection [35].

In summary, we have shown that the age stratified seroprevalence rates of dengue in suburban areas in Colombo have significantly increased over a 12 year period, possibly due to increase in transmission. We have also shown that JEV seropositivity is associated with a risk of hospitalization when infected with the DENV. It remains possible that this is explained by cross-reactivity between the assays, but it would be crucial to investigate this question further perhaps by using T cell or antibody responses to unique regions of DENV and JEV; and in particular to understand the mechanisms by which immune responses against JEV following vaccination or natural infection, might modulate clinical disease severity in subsequent dengue infections.
